# Reclassification of Kidney Clear Cell Carcinoma Based on Immune Cell Gene-Related DNA CpG Pairs

**DOI:** 10.3390/biomedicines9020215

**Published:** 2021-02-20

**Authors:** Qizhan Luo, Thomas-Alexander Vögeli

**Affiliations:** Department of Urology, RWTH Aachen University, University Hospital RWTH Aachen, Pauwelsstrasse 30, 52074 Aachen, Germany; qizhan.luo@rwth-aachen.de

**Keywords:** immune cell infiltration, CpGs (methylation sites) pair, kidney clear cell carcinoma, classification, subtype, mutation, copy number variation, tumor microenvironment, immune checkpoints, immunotherapy, inflammation, endothelial cells, fibroblast, PD-L1

## Abstract

Background: A new method was developed based on the relative ranking of gene expression level, overcoming the flaw of the batch effect, and having reliable results in various studies. In the current study, we defined the two methylation sites as a pair. The methylation level in a specific sample was subject to pairwise comparison to calculate a score for each CpGs-pair. The score was defined as a CpGs-pair score. If the first immune-related CpG value was higher than the second one in a specific CpGs-pair, the output score of this immune-related CpGs-pair was 1; otherwise, the output score was 0. This study aimed to construct a new classification of Kidney Clear Cell Carcinoma (KIRC) based on DNA CpGs (methylation sites) pairs. Methods: In this study, the biomarkers of 28 kinds of immune infiltration cells and corresponding methylation sites were acquired. The methylation data were compared between KIRC and normal tissue samples, and differentially methylated sites (DMSs) were obtained. Then, DNA CpGs-pairs were obtained according to the pairs of DMSs. In total, 441 DNA CpGs-pairs were utilized to construct a classification using unsupervised clustering analysis. We also analyzed the potential mechanism and therapy of different subtypes, and validated them in a testing set. Results: The classification of KIRC contained three subgroups. The clinicopathological features were different across three subgroups. The distribution of immune cells, immune checkpoints and immune-related mechanisms were significantly different across the three clusters. The mutation and copy number variation (CNV) were also different. The clinicopathological features and potential mechanism in the testing dataset were consistent with those in the training set. Conclusions: Our findings provide a new accurate and stable classification for developing personalized treatments for the new specific subtypes.

## 1. Introduction

Kidney clear cell carcinoma (KIRC) is the most prevalent, aggressive, and lethal type of kidney carcinoma [1,2,3]. Previous genomic studies of KIRC have displayed high molecular heterogeneity, and further categorization of these malignancies is urgently required to improve diagnosis and treatment schemes [3,4,5]. Tumor-infiltrating lymphocytes are the most broadly studied populations of tumor-infiltrating immune cells (TIIC) that play a vital role in the prognosis of KIRC [6]. Infiltrating CD4+ T cells can mediate renal cell carcinoma cell proliferation by regulating the TGFβ1/YBX1/HIF2α signal [7]. 

Recently, a study reported that diverse immunotherapy methods successfully treated a large number of fatal cancers [8]. The FDA has approved Nivolumab, a checkpoint blocker for renal cell carcinoma cell treatment, improving treatment prospects [9]. The combination therapy with bevacizumab and atezolizumab has revealed beneficial outcomes in the high PD-L1 expression population in a randomized trial [10]. A remarkable difference in the response rates to immunotherapy across individuals is that not all immunotherapy can successfully treat patients [11], and not all patients responded to immunotherapy.

The definition of batch effects is that non-biological factors in an experiment lead to changes in the data produced by the experiment. Because of laboratory conditions, reagent lots, and personnel differences, batch effects can lead to erroneous biological conclusions [12,13,14,15], and gene pairs can overcome the batch effects of various platforms [12,13,14,15]. Recently, a new method based on the relative ranking of gene expression levels was found to overcome the flaw of the batch effect, and yielded reliable results in several studies [14,15,16].

In a previous study, lung adenocarcinoma was classified into seven subtypes based on immune-associated gene pairs [17]. By contrast, our study has some differences. The first difference is that our analysis was based on immune-related CpGs (methylation sites) pairs. The second difference is that we analyzed the classification of KIRC via multi-omics. In fact, the classification of CpGs-pair subtypes may help to enhance the optimal scheme of KIRC patients that are responsive to immunotherapy. Our previous study reported that this method was successful in classifying bladder cancer into three clusters based on immune cell gene-associated CpG sites [18]. In the present study, we classified KIRC into three clusters based on the immune cell gene-associated CpGs-pair and analyzed the bio-mechanism according to the partial methods in our previous study. In our study, we defined the two methylation sites as a pair. The methylation level in a specific sample is subject to pairwise comparison to calculate a score for each CpGs-pair. The score was defined as a CpGs-pair score. Then, we classified the KIRC into three subgroups based on the CpGs-pairs.

## 2. Results

### 2.1. Classification Based on CpGs-Pair

**Figure 1:**
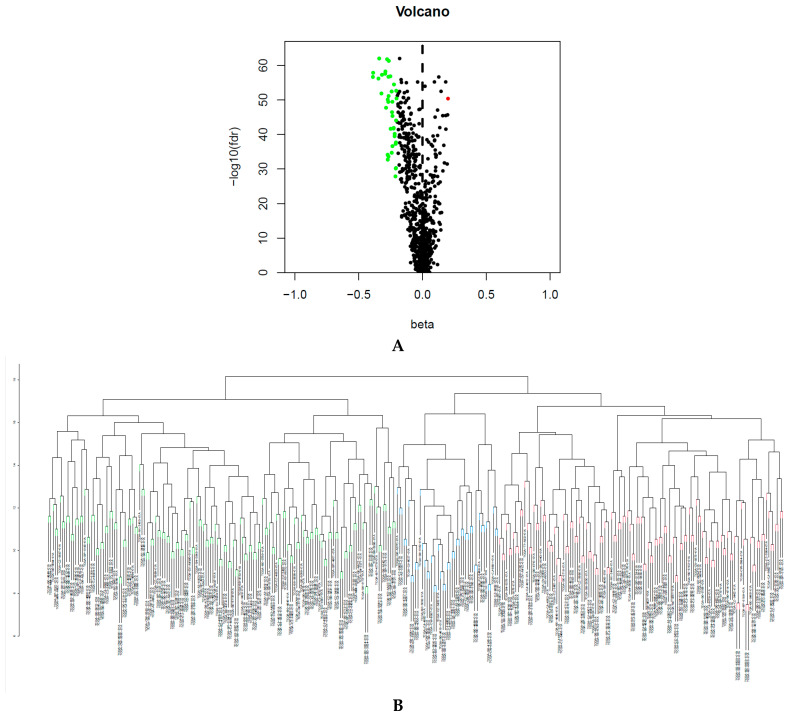

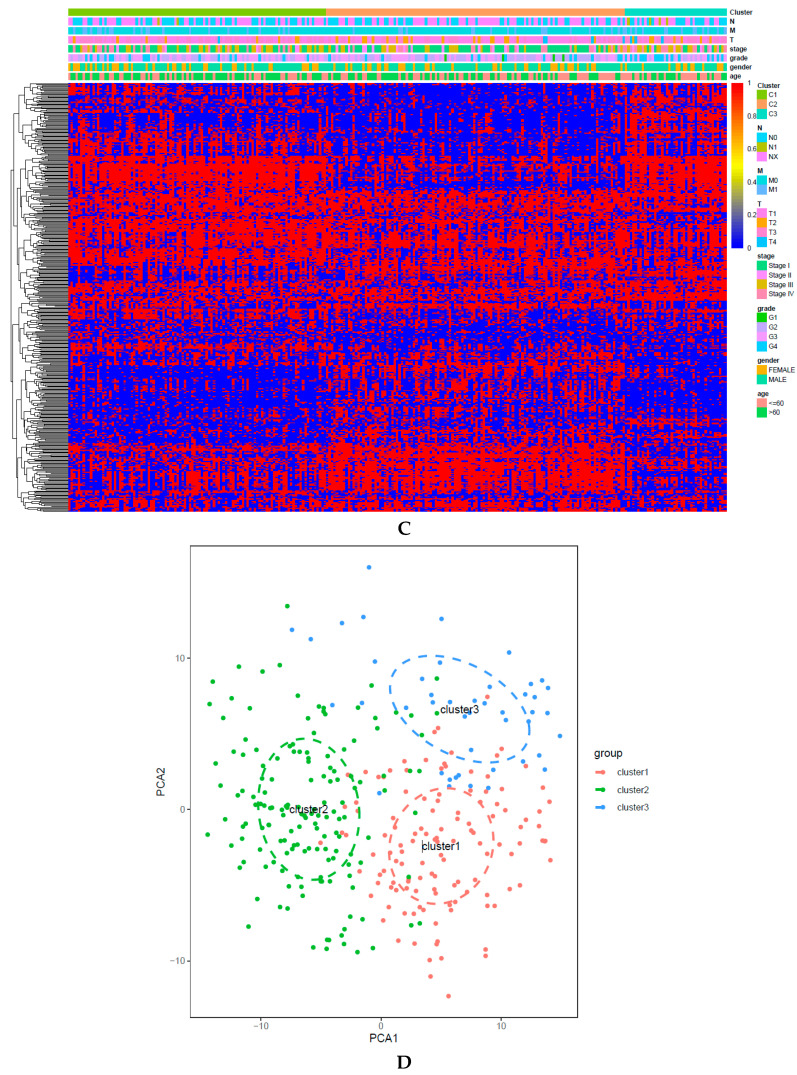
(**A**) Differentially methylated sites (DMSs) between normal samples and bladder cancer samples. (**B**) The consensus clustering of 441 CpGs-pairs was divided into three subgroups. (**C**) Heatmap of three clusters. (**D**) Principal component analysis (PCA) validated the stability of the classification.

We obtained 782 immune infiltration cell-related biomarkers from previous reports [19] and acquired 1138 corresponding immune cell biomarker-associated methylation sites. The false discovery rate (FDR) is used in multiple hypothesis testing to correct for multiple comparisons. FDR < 0.05 and |deltabeta value| > 0.2 were set as the parameters of infiltration. We identified 40 differentially methylated sites (DMSs) between 160 normal and 323 KIRC tissue samples with the Wilcoxon test (Figure 1A) in the training set. A total of 441 CpGs-pairs were obtained in the training set. The consensus clustering of 441 CpGs-pairs was clearly divided into three subgroups, as shown in Figure 1B. Figure 1C shows three distinct clusters. Principal component analysis (PCA) proved that the consensus classification was accurate and stable (Figure 1D). Cluster 1 contained 125 samples; cluster 2 contained 153 samples; and cluster 3 contained 45 samples.

### 2.2. Survival Rates and Clinicopathologic Features of CpGs-Pair Subtypes of KIRC

**Figure 2:**
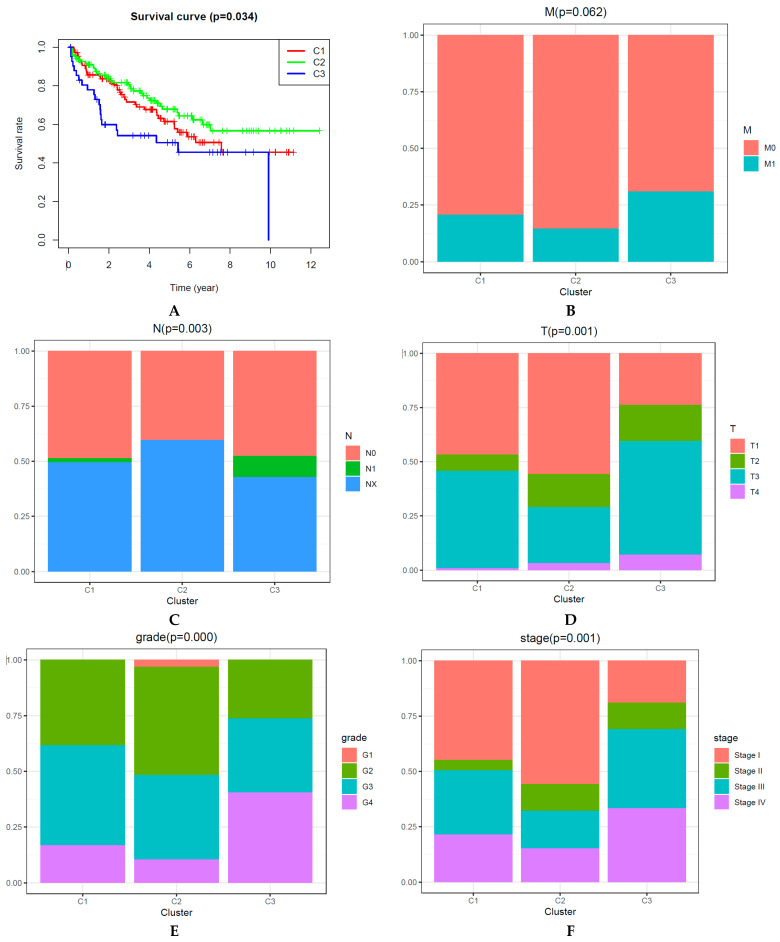

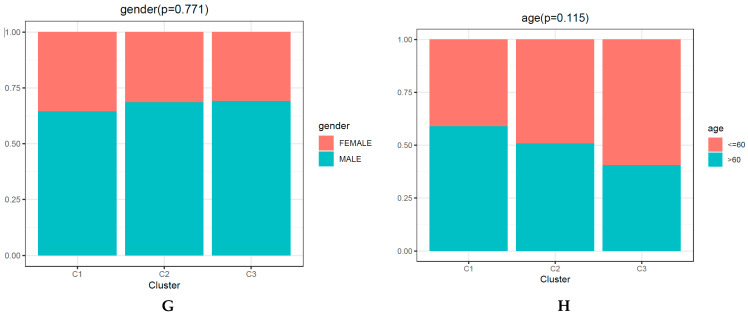
Main clinicopathological significances are different among three subgroups. (**A**) Overall survival curve for each DNA CpGs-pair subtype. (**B**–**H**) Clinicopathological features (M (metastasis) status, N (nearby lymph nodes) status, T (tumor) status, grade, stage, gender, age) among each DNA CpGs-pair subtype.

The Kaplan–Meier method was used to determine the overall survival (OS) curve of KIRC subgroups (Figure 2A). Cluster 2 had the best survival rate. Cluster 3 presented the poorest survival rate. Next, we compared survival rates between each pair of subgroups, and statistically different survival rates were only found between clusters 2 and 3 (Figure 2A). However, several previous publications showed that a remarkable difference between each pair of clusters was not mandatory [20,21,22,23]. Moreover, later analysis also showed that the clinical significance and the immune-related bio-mechanism among the three subgroups were different. Thus, KIRC was classified into three clusters.

In the present paper, the correlation between the main clinicopathological features and the subtypes is presented (Figure 2B–H). Excluding age, gender, and M (metastasis) status (*p*-value is 0.062), the three subgroups had significantly different clinicopathological features.

### 2.3. Identifying Distinct Methylation and Gene Expression Levels of Each DNA CpGs-Pair Subtype

**Figure 3:**
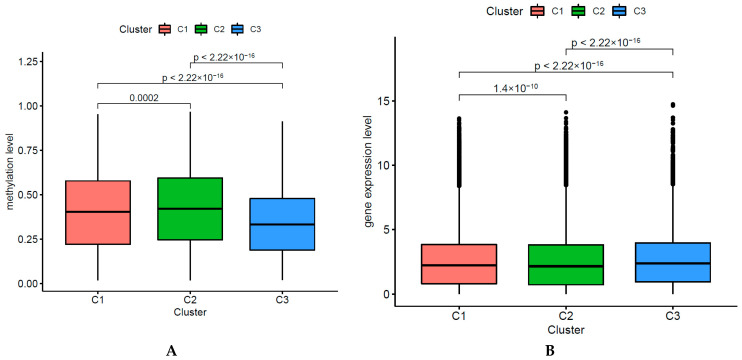
Distinct DNA methylation and gene expression level of each subtype. (**A**) Methylation level of each subtype. (**B**) Gene level of each subtype.

We identified the DNA methylation levels (40 DMSs) among the three DNA CpGs-pair subgroups and analyzed immune cell-associated gene-expression levels among those subgroups. The deferentially immune cell-associated methylation levels are presented, as shown in Figure 3A. Subgroups 1, 2, and 3 presented mid-range methylation, the highest methylation level, and the lowest methylation level, respectively. The three subgroups had significantly different DNA methylation levels (chi-square test, *p*-value < 0.001). Because we used the CpGs-pair scores to classify KIRC into three subgroups, and each subtype had half of methylated sites with higher methylation levels and half of methylated sites with lower methylation levels, the medium methylation levels among the three subgroups only had small disparities. We also found that the three subgroups had significantly different gene expression levels (chi-square test, *p*-value < 0.001) (Figure 3B).

In a comparison of CpGs-pairs of one cluster with the other two clusters (clusters 1, 2, and 3) (Wilcoxon test, FDR < 0.05, logFC > 0.2), a total of 148, 159, and 107 CpGs-pairs were significant CpGs-pair scores in subgroups 1, 2 and 3, respectively. 

In a comparison of the DNA methylation sites of one cluster with the other two clusters (clusters 1, 2, and 3) (Wilcoxon test, FDR < 0.05), a total of 11, 17, and 6 DNA methylations had significantly higher methylation levels in clusters 1, 2, and 3, respectively. 

### 2.4. Immune-Associated Mechanism of Classification 

**Figure 4:**
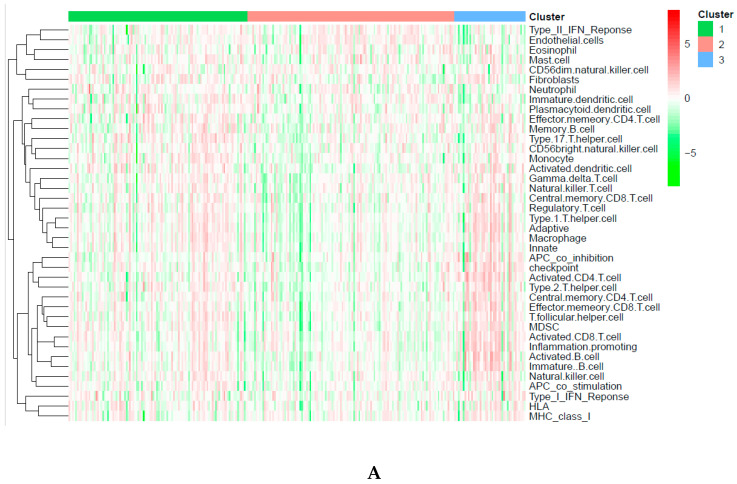

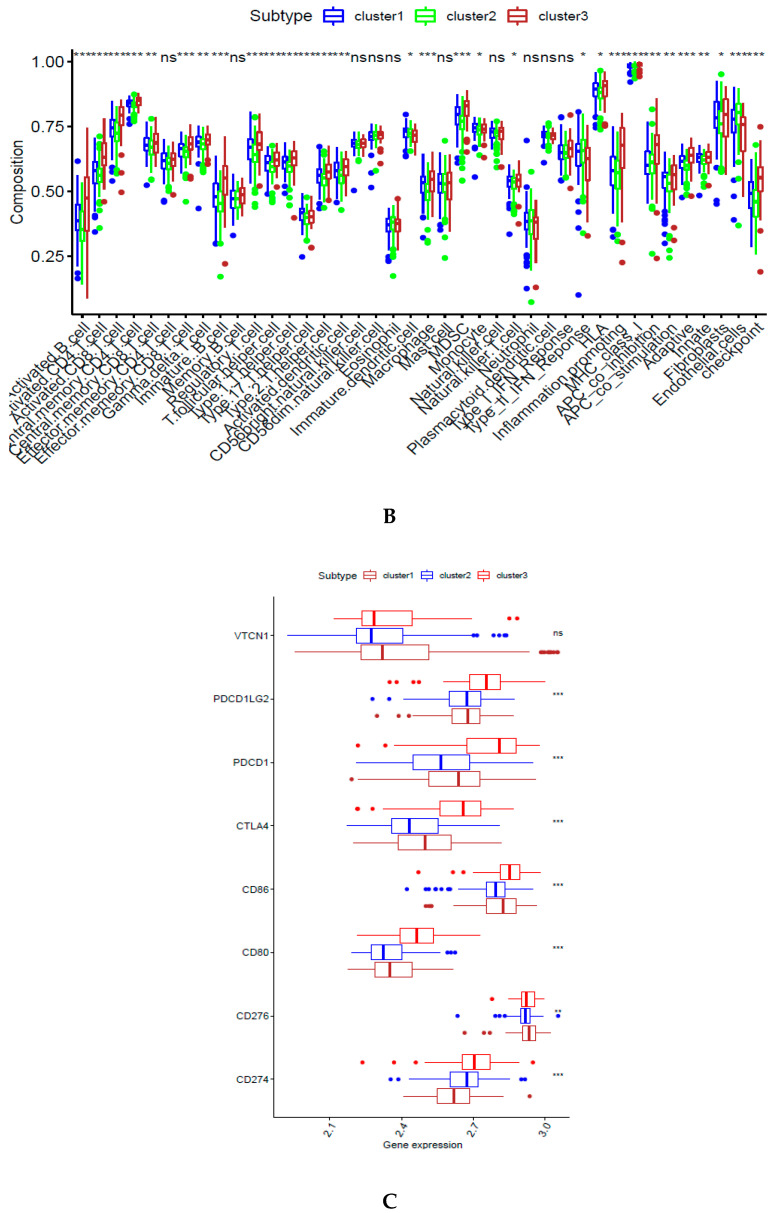

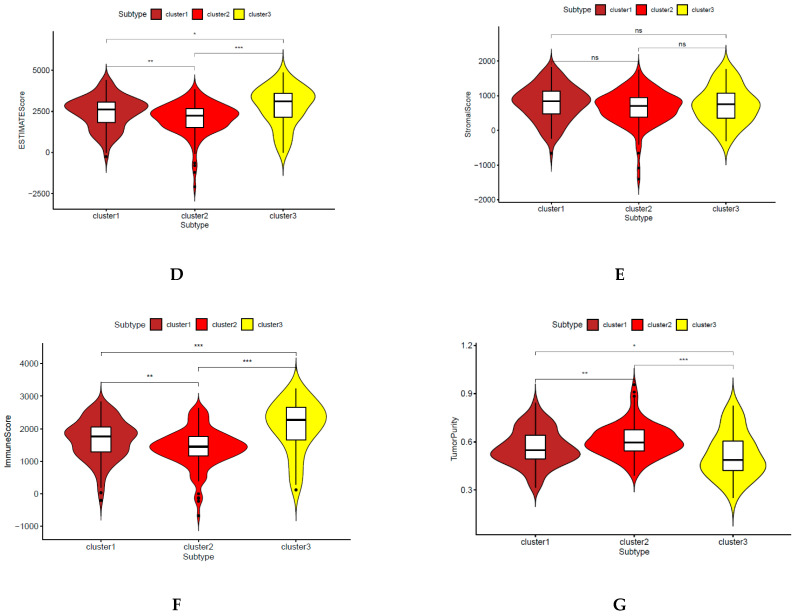
Immune status in different subgroups are different. (**A**) Immune infiltration level in different subgroups. (**B**). Immune infiltration cells in different subgroups. (**C**) Immune checkpoints in different subgroups. (**D**–**G**) Tumor microenvironment (TME). Three asterisks, two asterisks, and one asterisk represent a *p*-value less than 0.001, 0.01, and 0.05, respectively. NS represents no significance.

Immune infiltration was different among the three subgroups, as shown in Figure 4A. Cluster 2 was associated with the lowest immune infiltration. However, cluster 3 had the highest immune infiltration. Furthermore, 28 types of immune cells were different among these subtypes, including 19 types of immune cells with remarkable differences (Figure 4B). Seven immune checkpoints presented remarkable differences among these DNA CpGs-pair subtypes (Figure 4E).

### 2.5. Tumor Microenvironment (TME)

A previous study introduced the idea that the TME includes stromal cells, immune cells, and tumor cells [24]. The relationship is negative between the tumor purity and the sum of the stromal score plus immune score [24]. Subgroup 2 showed the lowest immune score, and ESTIMATE score, but the highest level of tumor purity, as shown in Figure 4D–G. However, subgroup 3 showed opposite results. The stromal score did not have a significant difference among three subgroups. The reason is that the classification was based on immune cell gene-related CpGs-pair scores and was not based on stromal cells.

### 2.6. Previous Classification

**Figure 5:**
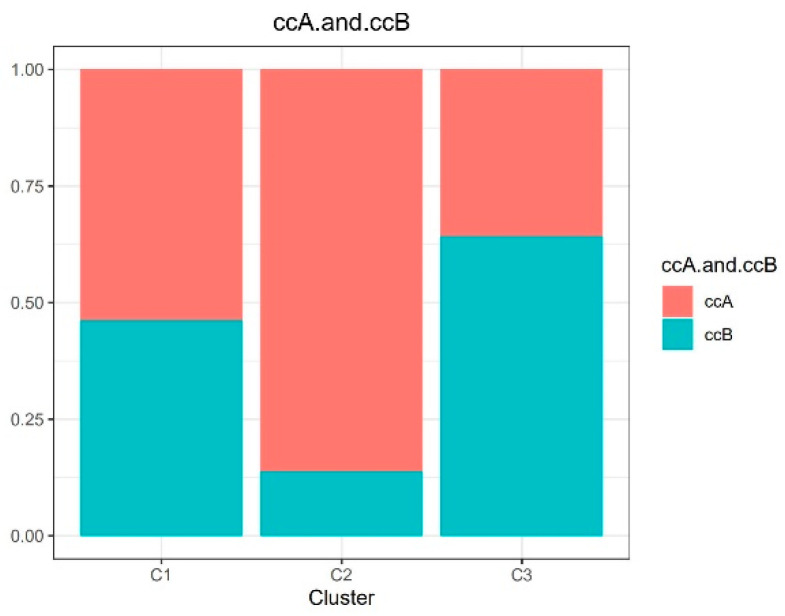
Comparison with previous classification.

A previous study reported that Clear Cell Renal Cell cCarcinoma was classified into two subtypes [25]: type A (ccA) and type B (ccB). Type A had a better survival rate than type B. The previous classification was based on single omics; however, our classification was based on multi-omics. In our study, we divided the KIRC into type A with 178 samples and type B with 93 samples. As shown in Figure 5, cluster 2 had the highest proportion of ccA, whereas cluster 3 had the lowest proportion of ccA. Similarly, in our study, cluster 2 had the best survival rates, but cluster 3 had the poorest survival rates. 

### 2.7. Validation of Classification Model

**Figure 6:**
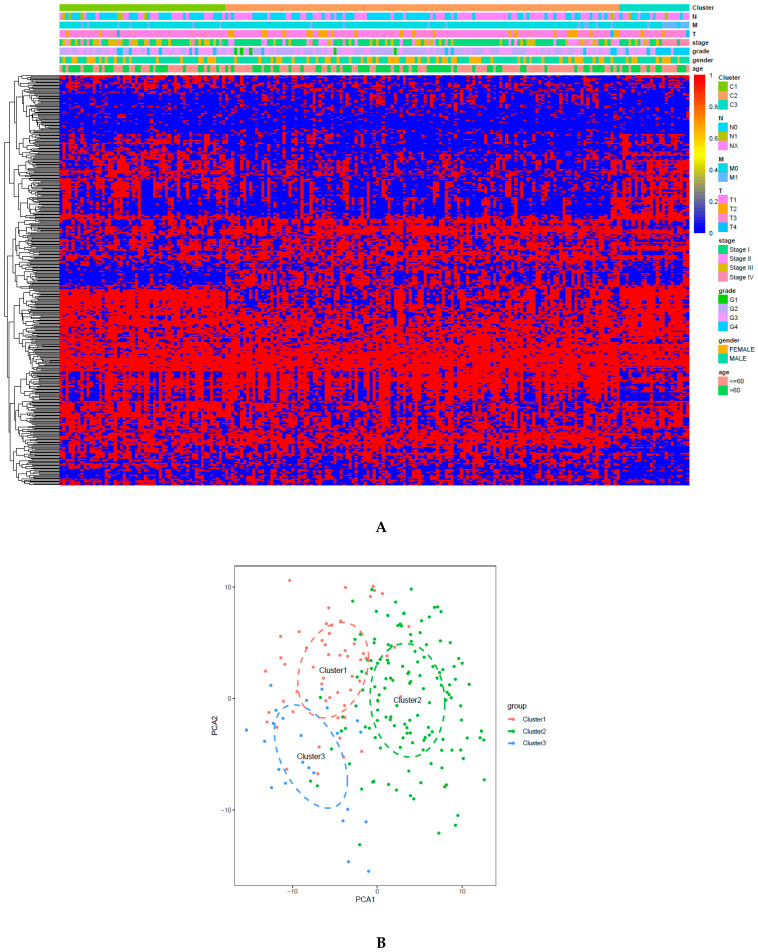

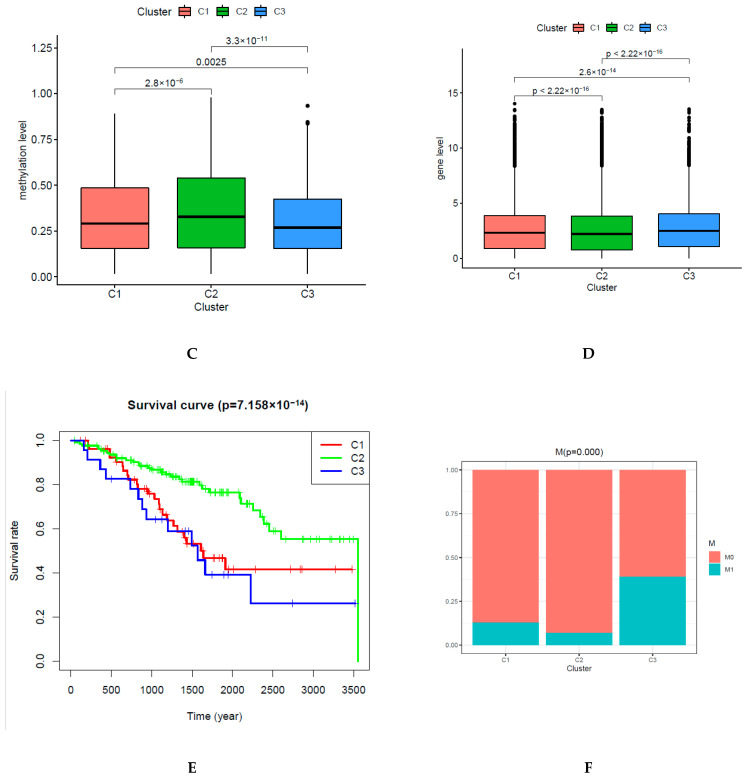

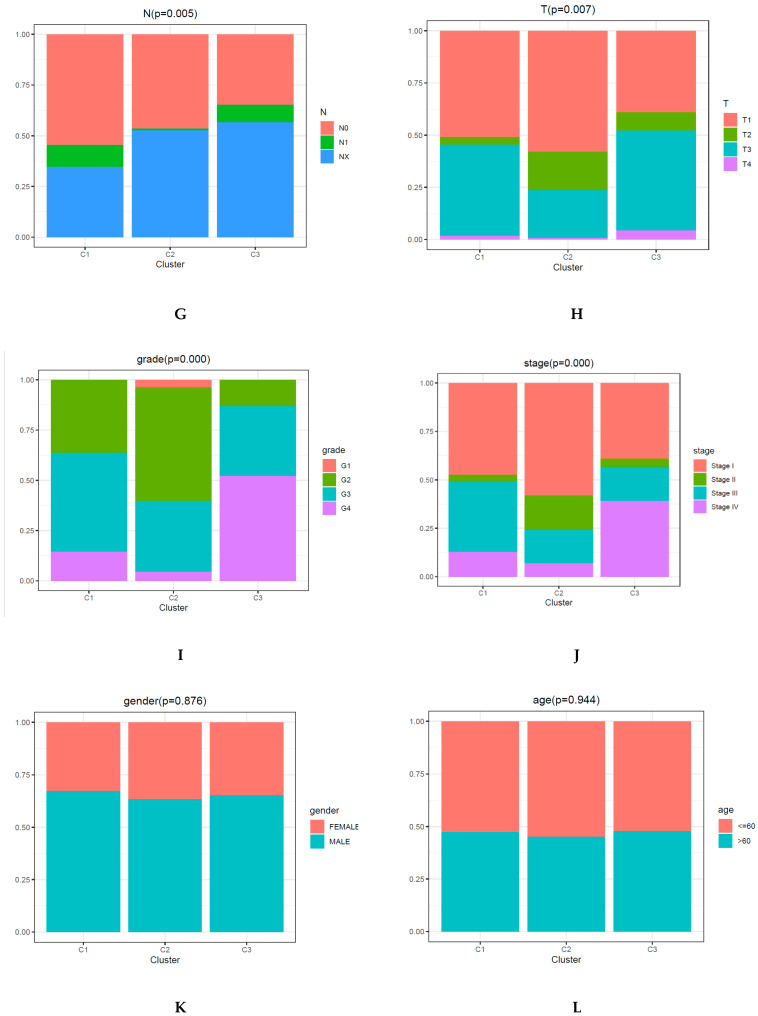
Classification according to CpGs-pair and clinicopathological features in the testing set is consistent with the training set and also different. (**A**) Heatmap of 441 CpGs-pairs. (**B**) PCA validated the stability of the classification. (**C**) Methylation level of each subtype. (**D**) Gene expression level of each subtype. (**E**) Overall survival curve for each DNA CpGs-pair subtype. (**F**–**L**) Clinicopathological features among each DNA CpGs-pair subtype.

**Figure 7:**
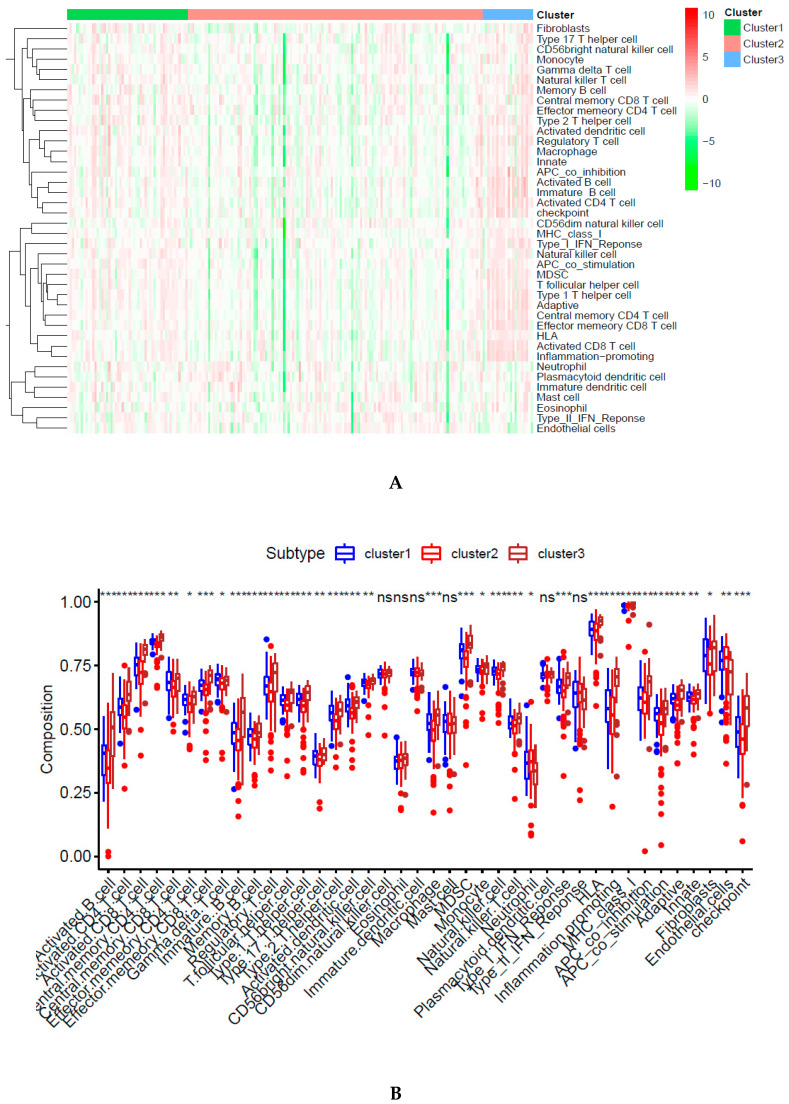

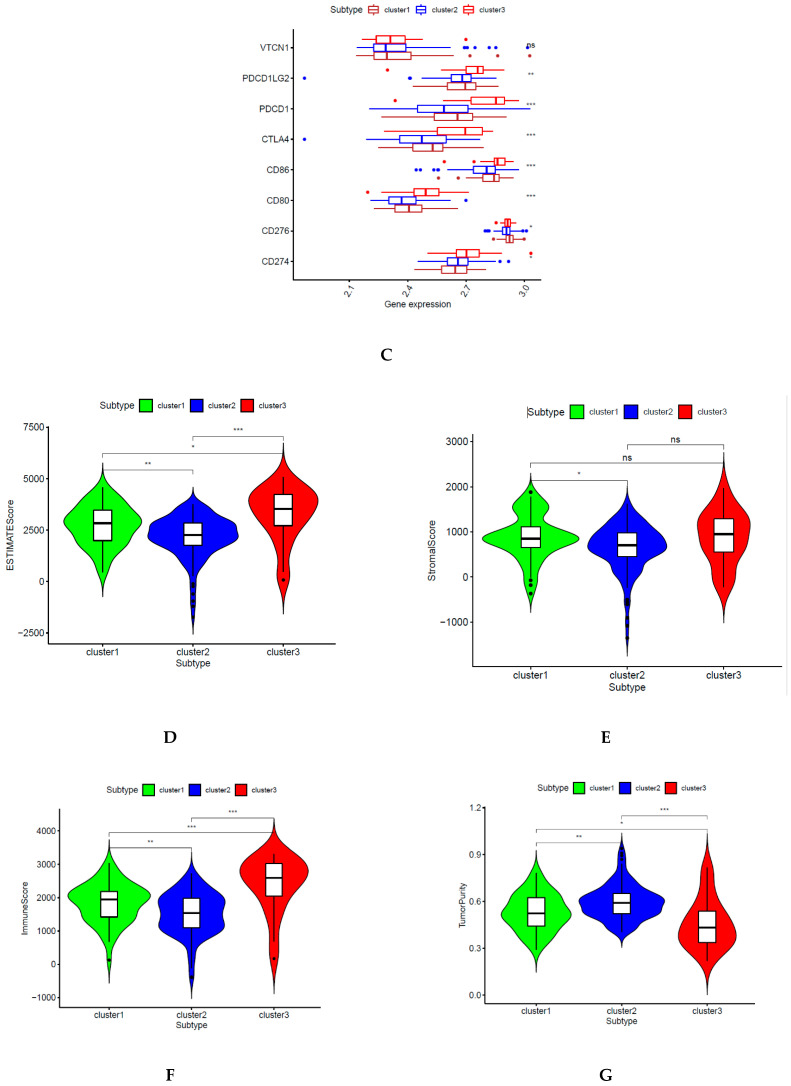
Immune status among different subgroups in the testing set is consistent with the training set and also different. (**A**) Immune infiltration level. (**B**) Immune infiltration cells. (**C**) Immune checkpoints. (**D**–**G**) TME. Three asterisks, two asterisks, and one asterisk represent a *p*-value less than 0.001, 0.01, and 0.05, respectively. NS represents no significance.

A support vector machine (SVM) was utilized to evaluate the performance of the classification of both sets and predict the DNA CpGs-pair subtypes in the testing set. We used the five-fold cross-validation method with the grid search method to obtain the optimum parameter (kernel: rbf; C: 10) and optimum SVM model (accuracy rate: 87.16%) based on 441 CpGs-pairs of the A group in the training set. Then, the optimum SVM model was tested in the B group and an accuracy rate of 97.53% was obtained. The samples in the testing set were classified into the corresponding subgroups based on the optimum SVM model. Cluster 1 contained 57 samples, cluster 2 contained 136 samples, and cluster 3 contained 24 samples. The function of the heatmap (Figure 6A) was annotated based on the classification of 441 CpGs-pair and clinicopathological staging in the testing set. PCA confirmed that the consensus classification was also accurate and stable (Figure 6B). Methylation and gene expression levels among those subgroups had significant differences (Figure 6C,D). Subgroups 1, 2, and 3 also presented mid-range methylation, the highest methylation level, and the lowest methylation level, respectively. These results were consistent with the training set, as shown in Figure 3. The relationship between methylation level and gene level was also positive. The Kaplan–Meier survival curve diagram revealed a prognosis of 3 subtypes (*p* = 7.158 × 10^−04^; Figure 6E). Based on our results, the prognosis of Clusters 3 was the worst, whereas that of Cluster 2 was the best. Figure 6F–L displays intracluster proportions of T status, N status, M status, grade, stage, age, and sex. Based on our results, excluding age and gender, the three subgroups had significantly different clinicopathological features. These results were consistent with the training set. Immune infiltration was analyzed in the testing set. In Figure 7A,B, cluster 3 was associated with high immune infiltration. Cluster 2 was associated with low immune infiltration. CD80, CD86, CD276, CD274, CTLA4, PDCD1LG2, and PDCD1 had significant differences across three subgroups (Figure 7C and Figure 4C). Subgroup 2 showed the lowest immune score and ESTIMATE score, but the highest level of tumor purity, as shown in Figure 7D–G. However, subgroup 3 showed the opposite results. These results were also consistent with the training set (Figure 4D,E). 

### 2.8. Genomic Alteration of Classification

**Figure 8:**
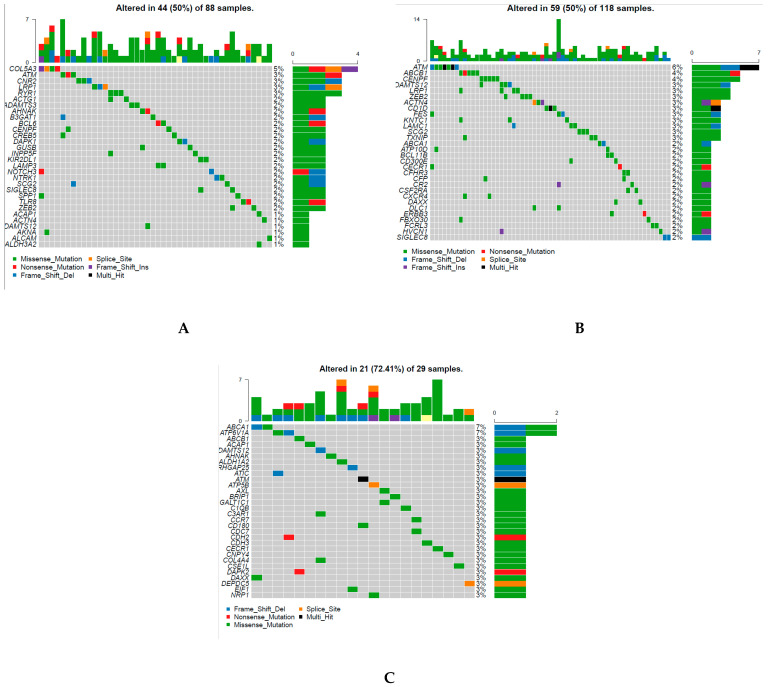

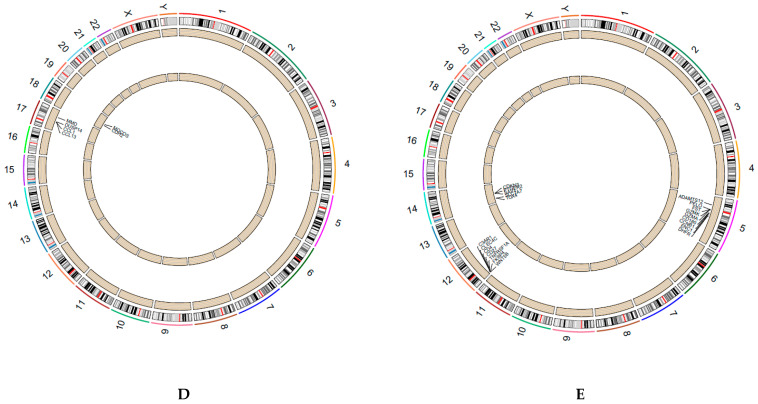
Genomic alteration of classification is different between each subtype. (**A**–**C**) 30 genes with the most frequent mutation in clusters 1, 2 and 3, respectively. (**D**,**E**) Copy number variation (CNV) in cluster 1 and cluster 2, respectively.

In each cluster, 30 genes with the most frequent mutation were obtained (Figure 8A–C), and 74 genes were identified from the 90 genes in three clusters. This implied that there was less overlap in the two groups based on the highest frequency of gene mutation (Figure 8A–C). Moreover, the somatic mutation of CD209 in subgroup 1 was remarkably more frequent than that in the other two subgroups. Similarly, the somatic mutation of ATP6V1A in cluster 3 was remarkably more frequent than that in the other two subtypes.

Figure 8D shows 20 genes with significant copy number gains and 2 genes with significant copy number losses in subgroup 1 compared with the other subgroups. Figure 8E shows 38 genes with significant copy number gains and 14 genes with significant copy number losses in subgroup 2 compared with the other subgroups.

## 3. Discussion

As a potentially promising biomarker, DNA methylation plays a significant role in diagnosis, tumor classification, and adjuvant therapies, according to several studies [26,27,28]. These studies indicate that DNA methylation plays a significant role in the modulation of the molecular structure and the expression of genes, and is correlated with many biological processes [3]. To understand the biological mechanism, help inform diagnosis and therapy, and promote prognoses, it is important to classify subtypes accurately. Several studies classified subtypes based on DNA methylation, including cervical tumors [29], glioblastomas [30], colon cancer [31], and bladder tumors [32]. According to the relative ranking of the gene expression level, a novel method was found to overcome the deficiency of the batch effect. It obtained reliable outcomes in several studies [14,15,16]. In a recent study, lung adenocarcinoma was classified into seven subtypes based on immune-associated gene pairs [17]. However, to the best of our knowledge, no study has constructed a new classification of KIRC based on immune-related CpGs pair profiles, which is what we achieved in this study.

In the present study, different CpGs-pair subtypes had different survival rates, as shown in Figure 2 and Figure 6. This may be due to the following: (1) Aberrant DNA methylation may result in a poor survival rate in tumor patients [33]. Low methylation values of SAT-α and L1 are correlated with poorer survival rates in patients with advanced gastric tumors [34]. A genome-wide low DNA methylation level is correlated with a poorer survival rate in early-stage colorectal tumor [35]. (2) High levels of immunosuppressive molecules can be expressed by cancer cells in the microenvironment to inhibit T cell proliferation and function while promoting tumor development and progression [36,37]. Previous studies showed that macrophages at high density in the microenvironment are associated with a poor survival rate in bladder cancer patients [38]. In our study, cluster 3 showed the lowest methylation level and the highest immune status, whereas cluster 2 showed the highest methylation level and the lowest immune infiltration. Thus, all previously mentioned factors might result in the poorest prognosis in cluster 3 and the best prognosis in cluster 2.

MMR (*MLH1, PMS2, MSH2, or MSH6*) deficiency, which is driven by inactivating methylation, was correlated with older age, advanced stage (II–IV), high grade of differentiation (G3), and larger tumor size [39]. Similarly, Cluster 3 showed the lowest methylation and several of the worst clinicopathological parameters, including more stage II and IV, more M1, more T3 and T4, more G4, and more N1. 

A previous study showed that two-thirds of the relationship between gene expression and DNA methylation in lung cancer was negative [40]. Similarly, another previous study showed that the high methylation cluster had low immune infiltration in breast tumors and skin cutaneous melanomas [41]. All of the above studies demonstrated a negative relationship between immune infiltration and methylation level. In the current study, cluster 3 with the lowest methylation levels had the highest immune infiltration; however, cluster 2 had the opposite outcome (Figure 3 and Figure 4).

RASAL1 silencing promotes kidney fibroblast and fibrogenesis activation through hypermethylation [42]. In the current study, cluster 2 with the highest methylation had the highest density of fibroblasts, as shown in Figure 3 and Figure 4.

Endothelial cells promote cancer cell intravasation and metastasis [43]. Upregulation of Notch1 of endothelia is correlated with poor prognosis in human cancer tissues, such as melanoma, serous ovarian carcinoma, lung adenocarcinoma, breast carcinoma, and colorectal carcinoma [44]. In the current study, cluster 2, with the lowest endothelial density, had a good survival rate; however, cluster 3 had the opposite results (Figure 3 and Figure 4).

A previous study reported that the immunity-high subgroup had high Human Leukocyte Antigen (HLA) expression, and the immunity-low subgroup had low HLA expression [45]. Similarly, the high immune cell infiltration subgroup also had high Type I IFN response, Type II IFN response, MIC class I, and APC [22,45,46]. The immune cell infiltration subgroup had opposite outcomes [22,45,46]. Our study found the same results as the above studies, as shown in Figure 5.

The negative or positive relationship between methylation levels and checkpoint expression levels depended on the specific CpG sites [47]. Furthermore, several previous studies showed that high immune infiltration is associated with high LD-1 (PDCD1) expression [22,45,46]. Moreover, two articles reported that one of the subgroups with high immune infiltration had PDCD1, PDCD1LG2, CD86, CD80, and CTLA4 overexpression [22,46]. In the current study, cluster 3 with high immune infiltration had high expression of PDCD1, PDCD1LG2, CD86, CD80, and CTLA4. 

Triple-negative breast cancers with PD-L1 overexpression responded robustly to immune checkpoint inhibitor therapy [48]. Similarly, higher PD-L1 expression was associated with better responses to Atezolizumab in the tumor-infiltrating leukocytes in bladder cancer [49]. In the present study, cluster 3 had PD-L1 overexpression and cluster 2 had the lowest PD-L1. This suggests that cluster 3 had a robust response to the PD-L1–Blocking Antibody; however, cluster 2 had the opposite response.

Although the molecular mechanisms are not yet understood [50,51,52,53,54,55], chronic inflammation plays a vital role in inducing abnormal methylation [50,51,52,53,54,55]. In our study, cluster 2, with high methylation, had low inflammation, whereas cluster 3, with low methylation, had high inflammation.

What is the correlation between mutation or CNV and DNA methylation? Previous research revealed that mutation might be promoted by hypomethylated blocks [56]. Mutations to thymine are caused by the methylation of cytosine [57]. However, another study showed that somatic mutations and differential promoter methylation interact with one another in head and neck tumors [58]. The genes of mutations were diverse among the three CpGs-pair subgroups. There was less overlap between the three subtypes (Figure 8A–C). Hypomethylated loci in tumors always coordinate with DNA break hotspots. Thus, this might result in copy number alteration [56]. As shown in Figure 8D,E, the genes with CNV were different between the two subgroups. This suggests that promising drug targets based on these CNV genes were different between the two subgroups. All of these genes with alteration show significant promise as drug targets.

Triple-negative breast cancers with PD-L1 overexpression responded robustly to immune checkpoint inhibitor therapy [48]. Similarly, higher PD-L1 expression has been associated with better responses to Atezolizumab in the tumor-infiltrating leukocytes in bladder cancer [49]. In the present study, cluster 3 had PD-L1 overexpression and cluster 2 had the lowest PD-L1. This suggests that cluster 3 had a robust response to the PD-L1–Blocking Antibody; however, cluster 2 had the opposite response. CD80, CD86, CD276, CD274, CTLA4, and PDCD1LG2 had significant differences across three subgroups (Figure 7C and Figure 4C). These checkpoints are potential treatment targets. 

A total of 11, 17, and 6 DNA methylations had significantly higher methylation levels in clusters 1, 2, and 3, respectively. These methylation sites were detected to differentiate the subtypes and show significant promise as drug targets. 

In conclusion, the successful classification of KIRC into three clusters was stable and accurate. The distribution of immune cells, stromal score, immune score, ESTIMATE score, tumor purity, checkpoints, HLA, endothelial cells, and inflammation were significantly different across the three clusters. The mutation and CNV were also different. The clinicopathological features and potential mechanism in the testing dataset were consistent with those in the training set. The study of the intratumoral immune microenvironment may provide a new perspective for therapy in KIRC.

## 4. Materials and Methods

### 4.1. Data Pre-Processing and Immune Cell-Associated Gene Selection

All of the methylation data were collected from the UCSC Cancer Browser (https://xenabrowser.net/datapages/). In total, 483 KIRC methylation datapoints were generated from Illumina Human Methylation 450 BeadChip, and 414 KIRC methylation datapoints were generated from Illumina Human Methylation 27 BeadChip. RNA-sequencing data (FPKM) from 611 KIRC tissue samples. The gene was kept when the medium gene expression level was great than 0. The Masked Somatic Mutation data (MuTect2. Somatic. Maf), the CNV data set (Masked Copy Number Segment, affymetrix snp 6.0), and corresponding clinical data were acquired from TCGA (https://cancergenome.nih.gov/). KIRC clinical data with follow-up times of more than 30 days were included. Samples with unknown grades, stages, T and M status were deleted. Thus, the clinical data contained 483 samples. The CNV data contained 1122 samples. Because our data were required directly from UCSC and TCGA websites, we strictly observed the publishing guidelines provided by the public databases; no requirement was needed for ethical approval.

The DNA methylation sites in promoter regions that were defined as from 0.5 kb downstream to 2 kb upstream of the transcription start sites strongly influenced gene expression [18,59,60]. We obtained 28 types of immune infiltration cell-associated genes from another study [19] (Table S1) and their corresponding methylation sites in promoter regions. Exclusions were based on the following probe criteria: (1) more than 70% of the sample data missed [61]; (2) probes on the X and Y chromosomes were removed [62]; (3) cross-reactive sites were excluded [62]. The k-nearest neighbors (KNN) imputation procedure was utilized to impute the remaining sites [31]. 

The samples were divided into a training set and a testing set. Data in the training set were from HumanMethylation 450 BeadChip, and data in the testing set were from HumanMethylation 27 BeadChip. The new method based on the relative ranking of the gene expression levels, overcame the flaw of batch effects, and had reliable results in several studies [14,15,16]. In the current study, the batch effects were not removed in data pre-processing.

### 4.2. Process of CpGs-Pairs

FDR less than 0.05 and a |deltabeta value| greater than 0.2 were set as the parameters of infiltration. DMSs were identified between KIRC and normal tissue samples with the Wilcoxon test. In previous research, the method used was as follows: if the first immune-related gene expression level was higher than the second immune-related gene expression level in a specific immune-related gene pair, the output score of this specific immune gene pair was 1; otherwise, the output score was 0 [14]. According to the above method, if the first immune-related CpG value was higher than the second in a specific CpGs-pair, the output score of this immune-related CpGs-pair was 1; otherwise, the output score was 0. If the score of an immune-related gene pair was 1 or 0 in more than 80% of the samples, then the immune-related gene pair was deleted from the training set [63]. 

### 4.3. Unsupervised Hierarchical Clustering 

Subtypes of KIRC were identified using unsupervised hierarchical clustering (complete linkage method, with cutree: (3) based on immune-related CpGs-pair scores with the “sparcl” package in R software obtained from a website (https://CRAN.R-project.org/package=sparcl). The Kaplan–Meier method was used to obtain the overall survival curve of the KIRC subgroups with the “survival” R software package. The classification was validated using PCA. The correlation between clinicopathology and the CpGs-pair clusters was analyzed. The statistical method was chi-square tests, and a *p*-value of <0.05 was considered statistically significant.

### 4.4. ssGSEA Based on Immune-Related Biomarker

We obtained the immune infiltration cell gene sets and the other immune-related gene sets from previous studies [19,45,64,65,66] (Supplementary Materials: Tables S1 and S2) and quantified the above gene sets using the ssGSEA that utilized the “GSVA” and “GSEABase” R packages to rank the genes based on their absolute expression. 

The enrichment score was calculated based on the integrated differences between the empirical cumulative distribution functions for the ranks of the genes [67]. Immune cells and the other immune-related gene set-rich scores were compared across subsets. Moreover, immune checkpoints [22,46] were compared across the subsets. The statistical method used was the Kruskal-Wallis test, and a *p*-value of <0.05 was considered statistically significant.

### 4.5. TME

We obtained the immune infiltration cell gene sets and the other immune-A previous finding showed that tumor stromal and immune cells in cancer tissues according to specific genes were estimated using an algorithm called the ESTIMATE algorithm [24], which was acquired online (https://sourceforge.net/projects/estimateproject/) [68]. The level of tumor stroma was predicted using the stromal scores. Similarly, the level of immune cells in tumor tissue was predicted using immune scores. The relationship between the combination of two types of scores and tumor purity was negative [68,69]. The immune scores, stromal scores, ESTIMATE scores, and tumor purity were compared across the subgroups. 

### 4.6. Validation of Classification Model

The classification model was validated using PCA. Several previous studies reported that the samples in training set were added labels with unsupervised hierarchical clustering. Then, a support vector machine (SVM) or Bayesian network classifier was used to obtain the optimum classification model from the training set and predict subtypes in the testing set [20,46,70]. The training set was randomly divided into two groups (the samples in A group: the samples of B group was 3:1). We used the five-fold cross-validation method and the grid search Method to obtain the optimum parameter and optimum SVM model based on 441 CpGs-pairs of the A group in the training set with Python software. Then, the optimum SVM model was tested in B group. Next, the samples in the testing set were divided into the corresponding subgroups based on the optimum classification model. The OS curve was constructed. The relationship between the biological characteristics of clusters and clinical information was shown via a bar plot. Immune infiltration and immune checkpoints were also analyzed in the testing set.

### 4.7. Genomic Alteration of Classification

The immune cell biomarker-associated mutation data (Table S1) were analyzed and visualized with the ‘maftools’ software package [71]. In a comparison of mutation data of one cluster with the other two clusters (clusters one, two, and three), the statistical method used was the chi-square test, and a *p*-value of <0.05 was considered statistically significant. Subsequently, we analyzed CNV data (Table S1) that were associated with the immune cell gene. The genomic identification of significant targets in a cancer (GISTIC) algorithm was utilized to identify the CNV genes [72,73]. The values 0.2 and −0.2 were used as the parameter thresholds for genomic gains and losses, respectively. Copy number alteration data in one group were compared with those in other two groups. A *p*-value of <0.05 was considered statistically significant. 

## Data Availability

Because our data were required directly from UCSC and TCGA websites, we strictly observed the publishing guidelines provided by the public databases; no requirement was needed for ethical approval.

## Supplementary Materials

The following are available online at https://www.mdpi.com/2227-9059/9/2/215/s1.

## Abbreviations

KIRC	Kidney Clear Cell Carcinoma
DNA CpGs	methylation sites-pairs
DMSs	differentially methylated sites
CNV	copy number variation
TME	tumor microenvironment
TIIC	Tumor-infiltrating immune cells
OS	overall survival
SVM	Support vector machine
PCA	Principal component analysis
